# Waterpipe smoking among university students in Hong Kong: a cross-sectional study

**DOI:** 10.1186/s12889-020-08686-6

**Published:** 2020-04-21

**Authors:** Jung Jae Lee, Yongda Wu, Man Ping Wang, Karly Cheuk-Yin Yeung, Janet Yuen-Ha Wong, Robert Smith

**Affiliations:** School of Nursing, Li Ka Shing Faculty of Medicine, The University of Hong Kong, 4/F, William M.W. Mong Block, 21 Sassoon Road, Pokfulam, Hong Kong

**Keywords:** Waterpipe smoking, Shisha, Hookah, University student, Prevalence, Young adult

## Abstract

**Background:**

Waterpipe smoking has gained global popularity in recent years, especially among young people. However, there is a lack of empirical investigation into waterpipe smoking in East Asia. This study aimed to investigate the demographical and psycho-social characteristics and patterns of waterpipe smoking (WPS) among university students in Hong Kong.

**Methods:**

A cross-sectional survey was conducted via online questionnaires administered to 1288 Hong Kong university students (mean age: 22.4 [SD = 3.8]). Logistic regressions were used to compute adjusted odd ratios (aOR) for waterpipe ever-smoking in relation to respondents’ characteristics. Moreover, multinomial logistic regression yielded adjusted RR (aRR) for four different smoking status (i.e., never, waterpipe-only, cigarette-only, and dual smoking) regarding the characteristics.

**Results:**

23.8% of participants reported having ever smoked a waterpipe (vs. cigarette ever-smoking: 21.1%). Factors including being female (aOR:1.57; 95% CI: 1.22–2.02), older age (≥24 years: 4.17; 1.35–12.93), frequent alcohol consumption (>monthly: 26.02; 10.91–62.09), and higher sensation-seeking behaviours (high level: 2.98;1.46–6.08) were associated with waterpipe ever-smokers. The study also identified that demographical and psycho-social characteristics were variably associated with students’ smoking status. Particularly, more frequent alcohol consumption was most significantly associated with waterpipe-only smoking (aRR:45.73; 95% CI:11.44–182.73) (vs. cigarette-only smoking: 3.01; 1.76–5.14).

**Conclusions:**

WPS is the most common form of tobacco smoking among university students in Hong Kong, and characteristics unique to the population were identified. There is no legislation of relevant policies on WPS despite its concerning significance in public health among young people, therefore immediate action to monitor and control WPS is needed in Hong Kong.

## Introduction

Waterpipe, also known as shisha and hookah, is a smoking device designed to generate tobacco smoke by placing lit charcoals on top of tobacco; the generated smoke then passes through water before the user inhales the smoke [1]. Contrary to the belief that waterpipe smoking (WPS) is safer than cigarette smoking (CS) because waterpipe smoke becomes purified as it passes through water, research has shown that waterpipe smoke contains just as many toxins as cigarette smoke. Therefore waterpipe smokers are exposed to the same health risks as cigarette smokers, such as acute and chronic respiratory and cardiovascular diseases [2–4]. However, compared to CS, waterpipe smokers are likely to inhale greater volumes of tobacco smoke due to longer smoking sessions and the use of charcoal, which further increases the levels of carbon monoxide and carcinogens in waterpipe smoke [5].

Despite the health risks, WPS has gained much popularity in many regions including the Eastern Mediterranean, North American, and European regions, especially with young people [6–9]. A recent systematic review reported that the prevalence of ever-WPS among youths ranged from 12.9 to 65.3% in the Eastern Mediterranean, from 3 to 44% in North America, and from 12.0 to 49.5% in Europe [9]. This is mainly due to several features of waterpipe smoking, including the introduction of sweet and aromatic flavours to tobacco for WPS [10, 11], peer influence [12], facilitated socialisation and social activity [2, 10, 11], the sense of belongingness and social inclusion [5, 10], online marketing strategies of WPS [13], the lack of waterpipe-specific regulations and policies [10, 11], and the misconception of WPS with regard to health [1]. In Hong Kong (HK), WPS is popular as evidenced by an increasing number of waterpipe establishments in busy city areas that are full of HK local customers, and the increasing media reports [14, 15]. Moreover, local studies [16, 17] reported that waterpipe was the second most popular tobacco product following cigarettes among adolescents in HK (1.2% current waterpipe smokers) and stressed the importance of continued surveillance of WPS in HK. However, there has been no WPS study on young adults in HK.

Despite the increasing popularity of WPS, published reports of the WPS global epidemiology [8–10, 18] have only covered the Eastern Mediterranean and North America regions, while the epidemiology in East Asia, including China, Korea, and Japan, which represent the second-largest population in the world following South Asia, is currently absent in the reports. It would be difficult to apply the research findings directly from the Eastern Mediterranean and North America regions to the East Asian regions, as the epidemiological characteristics and patterns of tobacco smoking including WPS differ among ethnic and/or socio-cultural groups [19–21]. Similarly, the World Health Organization [1] has urged researchers to conduct more WPS studies across regions with consideration for the cultural influence on WPS [1].

Moreover, WPS is influenced by psychosocial factors [5]. Specifically, sensation-seeking behaviour (SSB) is known as a predictor for problematic risk-taking behaviours such as substance use, smoking (including WPS), and problematic alcohol drinking [22, 23]. Loneliness is also known as a contributor to young people’s smoking behaviour [24]. Particularly, it has been hypothesised that WPS would likely be associated with loneliness as people can gain a sense of belonging and social inclusion through WPS [1, 2, 10]. However, there is a dearth of research exploring and defining the association between young people’s WPS and their levels of SSB and loneliness.

Therefore, this research on WPS in East Asia aimed to 1) investigate the demographical characteristics and patterns of WPS among university students in HK; 2) compare demographic characteristics between non-smokers, waterpipe-only ever-smokers, cigarette-only ever-smokers, and dual ever-smokers (i.e., waterpipe and cigarette); and 3) determine the associations of WPS and psychosocial factors.

## Methods

### Study design and sampling

A cross-sectional survey was conducted. All eight public universities funded by the University Grants Committee and form the major higher education institutions in HK were invited to collaborate with the study. One university declined the invitation due to their academic schedule. Each of the remaining seven universities’ Student Affairs or related administration department was asked to send the survey website link to enrolled students through registered student emails. This was sent as one bulk email to all students (total around 126,549 students, according to the universities’ reports) in April 2018 to reduce the burden of multiple bulk emails. The invitation email included general information about this study, such as details about the aim and incentive (i.e., Five participants randomly selected through lucky draw would receive HK$500 each [US$ 1 = HK$ 7.82] as a participation incentive). The participant inclusion criteria were: 1) currently registered as a student in a HK public university, 2) current HK resident, 3) English-speaking (main language of instruction in HK universities). The exclusion criteria were: 1) aged 17 years or younger, and 2) overseas exchange students.

Five thousand two hundred forty-three students visited the survey introduction website, and 1288 university students (331, 280, 247, 156, 105, 92, and 77 students from each university respectively) went on to participate in the survey after reading and agreeing to the consent form (response rate among all website visitors: 24.6%).

### Measurements

#### Demographic and WPS pattern

Demographic characteristics including gender, age, monthly household income, and degree pursued were collected. If participants had ever smoked a waterpipe, their patterns of WPS were surveyed (e.g., WPS initiation date, frequency of WPS during the last 12 months, length of an WPS session, reasons for WPS, places of WPS, WPS with alcohol drinking, and perceived addiction to WPS; Table 2). In addition to WPS, CS status (i.e., ever CS or never CS) and its initiation date were also noted. Participants also reported their frequency of alcohol consumption (never, once a month or less, more than monthly).

#### Brief sensation-seeking scale (BSSS)

The BSSS is shortened version of the 40-question Sensation Seeking Scale that measures the level of risk-taking behaviours, and has 8 questions [25]. The overall score of BSSS ranges from 8 to 40. Subjects with higher BSSS scores are more likely to seek risk-taking behaviours such as tobacco smoking than those of lower BSSS scores. The BSSS presented 0.76 internal consistency and reported construct validity [25]. It has been used for tobacco and alcohol studies with young adults [26], and has also been validated with the Chinese population [27]. We used the BSSS to measure the respondents’ risk-taking behaviours regarding WPS. The internal consistency of BSSS in this study was 0.82.

#### UCLA loneliness scale

We investigated the students’ level of loneliness using a short form of UCLA loneliness scale. The scale consists of three items ranging total score from 3 to 9, and reported 0.72 for internal consistency and both convergent and discriminant validity [28]. A score of ≥6 indicates that respondents feel lonely [29]. The UCLA loneliness scale was validated with Chinese adolescents [30]. The internal consistency of loneliness scale in this study was 0.81.

### Statistical analysis

Descriptive statistics were reported in proportions or means and standard deviations (SD). Median and interquartile ranges (IQR) were presented for skewed data. In those that reported trying WPS at least once (i.e., ever-users of waterpipe), descriptive characteristics of WPS behaviour were reported (i.e., patterns of WPS). To clarify differences between smoking behaviours, demographic and other descriptive statistics were compared between four smoking behaviour groups (never smoked, only ever smoked waterpipe, only ever smoked cigarettes, and ever smoked both cigarettes and waterpipe). Logistic regression yielded odd ratios (OR) and adjusted OR (aOR) for WPS (a binary variable). Multinomial logistic regression yielded risk ratios (RR) and adjusted RR (aRR) for four different smoking behaviours in relation to respondents’ characteristics. As WPS risk factors differed by gender in existing studies [11], we added interaction terms to test if gender modifies the association of alcohol consumption and psychosocial factors (i.e., SSB and loneliness) with ever use of waterpipe. Sensitivity analysis was also conducted through multiple imputation using chained equation to handle missing values [31].

## Results

Overall, 872 (67.7%) respondents were female, 897 (82.5%) were undergraduate students, 397 (42.4%) had a monthly household income between HK$20,000 and HK$49,999, and the mean age of respondents was 22.4 (SD = 3.8) years (Table 1). Among all respondents, 306 (23.8%) had ever used a waterpipe.

**Table 1:** Demographics of respondents according to waterpipe smoking status

	All respondents	Never smoked	Ever smoked	*p* value
**Total, n (%)**	1288	982 (76.2)	306 (23.8)	
**Gender, n (%)**				0.091
Males	416 (32.3)	315 (32.1)	101 (33.0)	
Females	872 (67.7)	667 (67.9)	205 (67.0)	
**Age, n (%)**				< 0.001
18–19 years	183 (14.3)	164 (16.7)	19 (6.2)	
20–21 years	463 (36.1)	364 (37.2)	99 (32.4)	
22–23 years	345 (26.8)	257 (26.3)	88 (28.8)	
24 year or older	293 (22.8)	193 (19.7)	100 (32.7)	
- Age, mean (SD)	22.4 (3.8)	22.1 (3.7)	23.2 (3.9)	< 0.001
- Initiation age, mean (SD)	–	–	20.2 (3.6)	
**Household income, n (%)**^a^				< 0.001
HK$19,999 or below	425 (45.4)	339 (47.3)	86 (39.3)	
HK$20,000 - HK$49,999	397 (42.4)	308 (43.0)	89 (40.6)	
HK$50,000 or above	114 (12.2)	70 (9.7)	44 (20.1)	
**Education level, n (%)**				0.217
Undergraduate	897 (82.5)	707 (83.3)	190 (79.8)	
Postgraduate	190 (17.5)	142 (16.7)	48 (20.2)	
**Alcohol consumption, n (%)**				< 0.001
Never	337 (26.2)	323 (32.9)	14 (4.6)	
Once a month or less	663 (51.5)	536 (54.6)	127 (41.5)	
More than monthly	288 (22.4)	123 (12.5)	165 (53.9)	
**Sensation-seeking behaviour, n (%)**				< 0.001
Low (8–18)	315 (24.5)	282 (28.7)	33 (10.8)	
Medium (19–29)	834 (64.8)	629 (64.1)	205 (67.0)	
High (30–40)	139 (10.8)	71 (7.2)	68 (22.2)	
**Loneliness, n (%)**				0.009
Not lonely	520 (40.4)	377 (38.4)	143 (46.7)	
Lonely (≥6)	768 (59.6)	605 (61.6)	163 (53.3)	

Among ever-waterpipe users (*n* = 306), more than half (56.2%) used it once a year or less, and the majority (96.7%) smoked waterpipe with friends (Table 2). Generally, each user spent less than HK$ 200 (42.8%) or HK$200 - HK$299 (33.7%) for one waterpipe session, and the length of each session was either less than 1 h (30.4%) or 1–2 h (47.7%). Waterpipes were usually available in both regular bars (66.3%) and waterpipe bars (57.3%), and were usually accompanied by alcohol consumption (86.9%). Users perceived that they were not addicted to WPS (95.8%) and could easily stop using it (score 10 of 10, IQR 9–10).

**Table 2:** Patterns of waterpipe smoking behaviours in respondents that have ever-smoked waterpipe

	Ever-smoked waterpipe (n = 306)
**Frequency of WPS in past 12 months, n (%)**	
Once a year or less	172 (56.2)
2–6 times	89 (29.1)
7–12 times	27 (8.8)
13–24 times	8 (2.6)
24–48 times	10 (3.3)
**Length of an WPS session, n (%)**	
Within one hour	93 (30.4)
1–2 h	146 (47.7)
2–3 h	57 (18.6)
3 or more hours	10 (3.3)
**Expense on WPS, n (%)**	
Less than HK$200 ^a^	131 (42.8)
HK$200-HK$299	103 (33.7)
HK$300-HK$399	49 (16.0)
HK$400-HK$499	11 (3.6)
HK$500 or more	12 (3.9)
**Reason for WPS, n (%)**^**b**^	
Socialising	253 (82.7)
Curiosity	215 (70.3)
Enjoy the taste/smell	147 (48.0)
Feeling relaxed	84 (27.5)
Good with alcohol	74 (24.2)
Less harsh than cigarette smoke	47(15.4)
Feeling high	36 (11.8)
Craving of waterpipe	5 (1.6)
Helping to quit smoking	3 (1.0)
Others	9 (2.9)
**Accompany with WPS, n (%)**^**b**^	
Friends	296 (96.7)
Family	10 (3.3)
Alone	7 (2.3)
Others	4 (1.3)
**Location of WPS, n (%)**^**b**^	
Regular bar	203 (66.3)
Waterpipe bar	175 (57.3)
Restaurant	29 (9.5)
Other home	20 (6.5)
Own home	16 (5.2)
Others	11 (3.6)
**Alcohol consumption with WPS, n (%)**	
Never	40 (13.1)
Rarely	67 (21.9)
Sometimes	79 (22.9)
Very often	49 (16.0)
Always	80 (26.1)
**Perceived addiction to WPS, n (%)**	
No	293 (95.8)
Yes	13 (4.2)
**Likelihood of stopping WPS, median (IQR)**	10 (9–10)

Key: ^a^ US$ 1 = HK$ 7.82; ^b^ respondents may give more than one response to item

The factors of being female (aOR1.57, 95% CI 1.22–2.02), older age (20–21 years: aOR 2.48, 95% CI 1.07–5.72; 22–23 years: aOR 3.05, 95% CI 1.43–6.09; ≥24 years: aOR 4.17, 95% CI 1.35–12.93), drinking (monthly or less: aOR 5.10, 95% CI 2.30–11.30; more than monthly: aOR 26.02, 95% CI 10.91–62.09), and medium (aOR 1.63, 95% CI 1.08–2.46) or high (aOR 2.98, 95% CI 1.46–6.08) SSBs were positively associated with ever use of waterpipe. Although the adjusted model was not significant, loneliness level was negatively associated with WPS in the crude model (OR 0.71, 95% CI 1.08–2.46) (Table 3). Additionally, the interactions between gender and alcohol consumption, SSBs, loneliness level were all not statistically significant (all *p* > 0.05, data not shown in tables).

**Table 3:** Factors associated with waterpipe smoking among respondents

	Odd Ratio (OR) (95% CI)	
	Crude	Adjusted ^b^
**Gender**		
Males	REF	REF
Females	0.96(0.78–1.17)	1.57(1.22–2.02)^***^
**Age**		
18–19 years	REF	REF
20–21 years	2.35(1.18–4.68)^*^	2.48(1.07–5.72)^*^
22–23 years	2.96(1.43–6.09)^**^	3.05(1.01–9.22)^*^
24 year or older	4.47(1.77–11.32)^**^	4.17(1.35–12.93)^*^
**Household income**^a^		
HK$19,000 or below	REF	REF
HK$20,000–$49,999	1.14(0.69–1.89)	0.87(0.52–1.45)
HK$50,000 or above	2.48(1.26–4.88)^**^	1.32(0.55–3.15)
**Qualification of study**		
Undergraduate	REF	REF
Postgraduate	1.26(0.83–1.90)	0.82(0.43–1.55)
**Alcohol consumption**		
Never	REF	REF
Once a month or less	5.47(2.78–10.77)^***^	5.10(2.30–11.30)^***^
More than monthly	30.95(16.33–58.67)^***^	26.02(10.91–62.09)^***^
**Sensation-seeking behaviour**		
Low (8–18)	REF	REF
Medium (19–29)	2.79(2.15–3.61)^***^	1.63(1.08–2.46)^***^
High (30–40)	8.18(6.13–10.93)^***^	2.98(1.46–6.08)^***^
**Loneliness**		
Not lonely	REF	REF
Lonely (≥6)	0.71(0.53–0.96)^*^	0.74(0.47–1.16)

Key: ^a^ US$ 1 = HK$ 7.82; ^b^ Adjusted for all variables listed in the table (i.e., gender, age, household income, qualification of study, alcohol consumption, sensation-seeking behaviour and loneliness); ^*^p < 0.05, ^**^p < 0.01, ^***^p < 0.001

Different demographical and other descriptive characteristics between the four smoking behaviour groups were identified, shown in Table 4. Moreover, some characteristics were differently associated with each group (Table 5). Waterpipe-only group showed similar associations with ever use of waterpipe, other than age variables. In cigarette-only users, being older and having higher SSBs, and being a postgraduate student (aRR 2.77, 95% CI 1.42–5.41) had increased risk of ever-cigarette use, while being female (aRR 0.62, 95% CI 0.45–0.84) and having a higher household income (HK$50,000 or above: aRR 0.34, 95% CI 0.16–0.72) had decreased risk of ever-cigarette use (all *p* < 0.05). Although alcohol consumption was positively associated with both cigarette-only and waterpipe-only users, much lower risk was identified with cigarette-only users (more than monthly: aRR 3.49, 95% CI 2.29–5.33). Moreover, feeling lonely was also associated with cigarette-only users in the crude model (RR 1.43, 95% CI 1.13–1.82). Older age, greater alcohol consumption, and higher SSBs also increased the aRRs in dual smokers (all *p* < 0.05). Results from the multiple imputations were generally similar (Additional files 1 and 2).

**Table 4:** Demographics of respondents according to smoking status

	Never smoked	Waterpipe only	Cigarette only	Dual smoking	*p* value
**Total, n (%)**	876 (68.0)	140 (10.9)	106 (8.2)	166 (12.89)	
**Gender, n (%)**					0.010
Males	277 (31.6)	33 (23.6)	38 (35.9)	68 (41.0)	
Females	599 (68.4)	107 (76.4)	68 (64.1)	98 (59.0)	
**Age, n (%)**					< 0.001
18–19 years	155 (17.8)	12 (8.6)	9 (8.6)	7 (4.2)	
20–21 years	344 (39.4)	54 (38.6)	20 (19.1)	45 (27.1)	
22–23 years	227 (26.0)	44 (31.4)	30 (28.6)	44 (26.5)	
24 year or older	147 (16.8)	30 (21.4)	46 (43.8)	70 (42.2)	
- Age, mean (SD)	21.9 (3.5)	22.3 (2.6)	24.1 (4.7)	24.0 (4.6)	< 0.001
- Initiation age, mean (SD)	–	19.9 (2.4)	17.2 (5.1)	20.5 (4.3)^b^/18.8 (4.0)^c^	
**Household income, n (%)**^a^					< 0.001
HK$19,999 or below	288 (45.5)	40 (39.2)	51 (60.7)	46 (39.3)	
HK$20,000 - HK$49,999	282 (44.6)	40 (39.2)	26 (31.0)	49 (41.9)	
HK$50,000 or above	63 (10.0)	22 (21.6)	7 (8.3)	22 (18.8)	
**Qualification of study, n (%)**					< 0.001
Undergraduate	652 (86.1)	100 (87.7)	55 (59.8)	90 (72.6)	
Postgraduate	105 (13.9)	14 (12.3)	37 (40.2)	34 (27.4)	
**Alcohol consumption, n (%)**					< 0.001
Never	301 (34.4)	5 (3.6)	22 (20.8)	9 (5.4)	
Once a month or less	477 (54.5)	76 (54.3)	59 (55.7)	51 (30.7)	
More than monthly	98 (11.2)	59 (42.1)	25 (23.6)	106 (63.9)	
**Sensation-seeking behaviour, n (%)**					< 0.001
Low (8–18)	264 (30.1)	17 (12.1)	18 (17.0)	16 (9.6)	
Medium (19–29)	560 (63.9)	101 (72.1)	69 (65.1)	104 (62.7)	
High (30–40)	52 (5.9)	22 (15.7)	19 (17.9)	46 (27.7)	
**Loneliness, n (%)**					0.021
Not lonely	344 (39.3)	68 (48.6)	33 (31.1)	75 (45.2)	
Lonely (≥6)	532 (60.7)	72 (51.4)	73 (68.9)	91 (54.8)	

Key: ^a^ US$ 1 = HK$ 7.82; ^b^ Waterpipe; ^c^ Cigarette

**Table 5:** Factors associated with smoking status among respondents

	Waterpipe only		Cigarette only		Dual smoking	
	Relative risk ratio (RR) (95% CI)		Relative risk ratio (RR) (95% CI)		Relative risk ratio (RR) (95% CI)	
	Crude	Adjusted ^b^	Crude	Adjusted ^b^	Crude	Adjusted ^b^
**Gender**						
Males	REF	REF	REF	REF	REF	REF
Females	1.50(1.05–2.14)^*^	1.92(1.14–3.22)^*^	0.83(0.64–1.07)	0.62(0.45–0.84)^**^	0.67(053.-0.83)^***^	1.11(0.85–1.45)
**Age**						
18–19 years	REF	REF	REF	REF	REF	REF
20–21 years	2.03(1.18–3.48)^*^	1.91 (0.91–3.97)	1.00(0.61–1.65)	0.87(0.43–1.76)	2.90(1.04–8.06)^*^	3.59(1.03–12.54)^*^
22–23 years	2.50(1.50–4.17)^***^	2.48(1.09–5.65)^*^	2.28(0.88–5.87)	2.19(1.20–3.98)^*^	4.29(1.35–13.68)^*^	5.22(0.88–31.10)
24 year or older	2.64(1.07–6.48)^*^	2.53(0.94–6.82)	5.39(2.72–10.67)^***^	2.26 (1.26–4.07)^**^	10.54(3.51–31.69)^***^	9.28(2.03–42.44)^**^
**Household income**^a^						
HK$19,000 or below	REF	REF	REF	REF	REF	REF
HK$20,000–$49,999	1.02(0.59–1.78)	0.78(0.47–1.29)	0.52(0.36–0.76)^**^	0.51(0.37–0.72)^***^	1.09(0.61–1.95)	0.77(0.43–1.38)
HK$50,000 or above	2.51(1.25–5.07)^*^	1.40(0.55–3.55)	0.63(0.35–1.13)	0.34(0.16–0.72)^**^	2.19(1.08–4.41)^*^	0.83(0.28–2.44)
**Qualification of study**						
Undergraduate	REF	REF	REF	REF	REF	REF
Postgraduate	0.87(0.42–1.78)	0.71(0.26–1.96)	4.18(3.25–5.36)^***^	2.77(1.42–5.41)^**^	2.35(1.60–3.44)^***^	1.35(0.64–2.84)
**Alcohol consumption**						
Never	REF	REF	REF	REF	REF	REF
Once a month or less	9.59(3.75–24.56)^***^	13.98(4.42–45.24)^***^	1.69(1.16–2.46)^**^	1.70(0.97–2.99)	3.58(1.64–7.77)^**^	2.59(1.11–6.01)^*^
More than monthly	36.24(15.03–87.38)^***^	45.73(11.44–182.73)^***^	3.49(2.29–5.33)^***^	3.01(1.76–5.14)^***^	36.17(18.75–69.78)^***^	25.66(10.74–61.32)^***^
**Sensation-seeking behaviour**						
Low (8–18)	REF	REF	REF	REF	REF	REF
Medium (19–29)	2.80(1.87–4.19)^***^	1.72(1.13–2.62)^*^	1.81(1.13–2.89)^*^	1.36(0.63–2.93)	3.06(2.47–3.80)^***^	1.64(1.02–2.64)^*^
High (30–40)	6.57(4.29–10.06)^***^	2.79(1.49–5.24)^**^	5.36(3.33–8.63)^***^	3.58(1.38–9.27)^**^	14.60(9.28–22.96)^***^	4.82 (1.81–12.88)^**^
**Loneliness**						
Not lonely	REF	REF	REF	REF	REF	REF
Lonely (≥6)	0.68(0.43–1.10)	0.77(0.52–1.16)	1.43(1.13–1.82)^**^	1.37(0.76–2.49)	0.78(0.59–1.04)	0.78(0.42–1.44)

Key: ^a^ US$ 1 = HK$ 7.82; ^b^ Adjusted for all variables listed in the table (i.e., gender, age, household income, qualification of study, alcohol consumption, sensation-seeking behaviour and loneliness); ^*^p < 0.05, ^**^p < 0.01, ^***^p < 0.001

## Discussion

To the best of our knowledge, this study offers the first evidence of the demographical characteristics and patterns of WPS in East Asia as well as the association of WPS with psychosocial factors and alcohol consumption among university students in multiple institutions in HK.

### Demographic characteristics of WPS

This research reported that the prevalence of ever-WPS was 23.8%, while the prevalence of ever-tobacco smoking (i.e., WPS and/or CS) was 32.0% among the respondents in HK. The ever-WPS prevalence in United States (US) studies ranged between 15.1 and 41% (mean: 30.3%) among university students [11] and 3 and 44% (mean 18.2%) among youth - defined by the United Nations as persons aged 15–24 years [9]. In some Middle Eastern countries like Iran, where WPS is more socially acceptable than in other regions, ever-WPS prevalence rates were higher than that of US studies (42.5% in Iran) [32]. Although the prevalence of WPS among university students in US was slightly higher than that of HK, WPS in the US was the second most popular form of smoking after CS [11]. In contrast, we reported that the prevalence of ever-WPS was higher than the ever-CS prevalence (21.1%: cigarette-only smoking 8.2% and dual smoking 12.9%), which is similar to the rates commonly reported in Middle Eastern studies such as Lebanon [33], Jordan [34] and Syria [35]. Due to governmental and social efforts toward tobacco control, HK achieved the lowest daily CS prevalence ever (10.0%) in 2017 [36], and has one of lowest prevalences of CS in the world [37]. However, WPS control has received less attention, and tobacco control counterparts have been less aware of the popularity of WPS as an alternative to CS in HK [16]. Such conditions result in a higher WPS prevalence than CS among university students in HK.

Female CS prevalence in HK has consistently been lower than most developed Western countries [37]. Li, et al. [38] attributed this to the strong social bias regarding female smokers in HK. We also identified that females are negatively associated with cigarette-only smoking. Being female, however, was positively associated with waterpipe smoking. It is also contrary to the existing WPS studies in both Western and Middle East countries that reported male students being more likely to smoke waterpipe than female students in the same countries [11, 32, 39]. It can be deduced that different social circumstances and dynamics surrounding WPS and CS (e.g., social acceptance of female WPS) would influence female WPS prevalence. Qualitative approaches will be required to identify the dynamics that are absent in the current knowledge base.

### Psychosocial characteristics of WPS

Socialisation was identified as the top reason for WPS in US (56%) and Iran (65.3%) studies [40, 41]. In this study, the major motives for WPS were generally similar to the above studies, namely socialisation (82.7%). It was also established that WPS is a social activity as a significant number of waterpipe users smoked waterpipe with others. An Iranian study demonstrated that university students smoked waterpipe with not only their friends (63%), but also siblings (61.9%) and parents (58.4%) [32]. The students in HK, however, had mostly smoked waterpipe with their friends or acquaintances (96.7%), rather than with their family (3.3%) to seek socialisation. Chinese culture is more collectivistic than their more individualistic Western counterparts [42]. In a collectivistic culture, engaging in a group activity plays an important part to one’s relationships with others within the group and their sense of belonging to the group [43]. This can be interpreted to indicate that HK students would more strongly seek membership within a peer group they are already affiliated to due to the collectivistic culture in HK. Additionally, according to collectivistic cultural values and the importance of social hierarchy, they would be reluctant to smoke WPS with their family members because they (particularly female waterpipe smokers) believe that smoking with their parents or with persons of higher social positions would be immoral and impudent [44, 45].

Similar to another study [46], we identified that cigarette users were positively associated with a perceived loneliness level. However, the ever-WPS group reported a negative association with loneliness level and the waterpipe-only group presented the lowest proportion of loneliness in this research. Primack, et al. [47] found that WPS was less associated with mental health problems (e.g., depression and anxiety) than CS. As such waterpipe smokers may feel less lonely than cigarette smokers because WPS is a social activity. We also identified that all smoking forms had positive associations with SSB, but that dual smokers showed the highest SSB. Heinz, et al. [5] asserted that one SSB is often related to other SSBs. WPS studies [17, 32, 39, 48, 49] found that WPS is significantly associated with CS or cigarette smoking susceptibility, as one tobacco smoking behaviour can be the gateway to smoking other types of tobacco. As the initiation age of cigarette smoking (i.e., 18.8 years) was earlier than waterpipe smoking (i.e., 20.5 years) in the dual smoking group in this study (Table 4), CS may play a gateway role in WPS. Further research is required to examine the relationship, however.

In addition to CS, a study reported that alcohol consumption was associated with WPS [39]. This is in parallel with a finding from this research, that the majority of ever-waterpipe smokers reported that they tended to smoke waterpipe while also drinking alcohol. The most popular sites for WPS among the ever-waterpipe users in HK were bars (regular bars: 66.3% and waterpipe bars: 57.3%). Particularly, waterpipe-only users showed much higher associations with alcohol consumption than those in the cigarette-only group. Existing studies indicated that the combination of CS with alcohol drinking will lead to increased risk of brain damage and increased mortality rate [50–54]. As waterpipe smoke contains as many toxins, it can be deduced that WPS with alcohol drinking may have a similar or more serious health impact than CS with alcohol drinking. However, to date, only a few studies have explored the relationship between WPS and alcohol consumption. Further WPS studies that focus on alcohol drinking are also recommended.

In HK bars, including those specialising in waterpipe, customers must order at least one item from the food and beverage menu in addition to the waterpipe. Therefore, the total cost of a WPS session is usually higher than the cost of a single waterpipe. We found that most ever-waterpipe smokers spent HK$200 (approximately US$26) or more for a single WPS session, which is more expensive than one pack of cigarettes (e.g., 1 pack of Marlboro cigarettes = HK$59) and considerably expensive for students. Corresponding to this, we also found that a high household income (HK$50,000 or above) was more positively associated with WPS (i.e., ever-waterpipe and waterpipe-only user groups). Conversely, high household income was negatively associated with the cigarette-only user group. However, a US study [11] argued that WPS is relatively inexpensive, which can be attributed to the difference in patterns of WPS between HK and other counties. Lipkus, et al. [6] reported that university students in the US typically smoked waterpipe at home or in student accommodations (46.4%), while only 5.2 and 6.5% of the HK students in this research smoked waterpipe at their own or others’ homes, respectively. As HK students mostly smoked waterpipe at bars or restaurants, they get charged per session and inevitably spend more money than US students.

Lastly, 96% of the respondents of this research who had ever smoked waterpipe in this research perceived the addiction risk of WPS positively. They were also confident about their ability to stop WPS as they wished. A literature review [18] reported that high proportions of waterpipe smokers in universities in both the Middle Eastern and Western countries (ranging between 79 and 98%) were highly confident about stopping WPS at any time they want, which is similar to the finding in this research. However, WPS is actually associated with a high risk of addiction [55], due to highly-addictive substances like nicotine in waterpipe tobacco and smoke [56, 57]. Despite the smokers’ confidence in being free from WPS addiction, they are undoubtedly exposed to a high chance of addiction.

### Implication

We have discovered that HK is not free from the global popularity of WPS (23.8% waterpipe ever smoking among HK university students). Thus, there is an urgent need to monitor and control the WPS trend within the HK population.

As most students initiated WPS during their university education (mean age: 20.2 years), WPS prevention education including education on WPS health risks should be developed and delivered to the students upon entry to university or at pre-university level. Moreover, given the association of WPS with CS and alcohol drinking, the existing CS and alcohol drinking prevention and cessation programme will need to be reviewed to include further information on the compounded health risks with the addition of WPS.

We also identified that bars were the most popular place for WPS. Particularly, the popularity and demand of waterpipe in HK can be inferred, as students can easily smoke waterpipe in mainstream bars in addition to waterpipe bars in HK. However, specific regulations for WPS in the bars are absent in HK and many other countries [1]. More seriously, most bars in HK allow indoor WPS, although it is illegal. Due to the indoor WPS, waterpipe smokers and staff in the bars are exposed to serious health conditions due to first-, second-, and third-hand smoking. Hence, the government’s surveillance of indoor WPS should be reinforced.

### Limitation

This research has several limitations. We adopted a cross-sectional survey; hence, causality should not accordingly be inferred in the results. We recruited participants from 7 universities in HK, but the participants were not necessarily a representative sample of university students or all young adults. We conducted an online survey by sending a bulk invitation to the students’ university email accounts. This method can cause the issue of multiple responses from one participant. To address multiple responses, we collected the first four digits of each respondent’s national identification number. Moreover, sampling bias can be caused by the online survey method. Therefore, further studies recruiting more representative sample participants from the same population as this study will be useful to warrant our findings [58]. The results were based on participants’ self-report, which would be subject to social desirability and recall biases. Despite these limitations, the findings of this research will provide important information regarding the WPS situation in East Asia to guide further WPS studies.

## Conclusion

Our findings highlighted that WPS is the most common form of tobacco smoking among university students in HK and has unique demographical and psychosocial characteristics that differ from those of the countries in which WPS is popular.

Because WPS among students has become a significant public health concern, policymakers, healthcare professionals, and researchers should take immediate action to extensively monitor and control this practice. As the demographical characteristics and patterns of WPS can be differentiated by socio-cultural group, more local and international studies are also required for the development of more effective strategies to address WPS.

## Supplementary information

**Additional file 1.** Title of data: Multiple imputation analyses of ever-use of waterpipe. Description of data: Sensitivity analysis of ever-use of waterpipe was conducted through multiple imputation using chained equation to handle missing values.

**Additional file 2.** Title of data: Multiple imputation analyses of smoking status. Description of data: Sensitivity analysis of smoking status was conducted through multiple imputation using chained equation to handle missing values.

## Data Availability

The datasets generated and analysed during the current study are not publicly available because the datasets are currently used for another project, but are available from the corresponding author on reasonable request.

## Abbreviations

aOR: Adjusted odd ratios
aRR: adjusted risk ratios
BSSS: Brief sensation-seeking scale
CI: Confidence Interval
CS: Cigarette smoking
HK: HongKong
IQR: Interquartile ranges
OR: Odd ratios
RR: Risk ratios
SSB: Sensation-seeking behaviour
SD: Standard deviations
US: United States
WPS: Waterpipe smoking
