# Supplementary material for: Waterpipe smoking among university students in Hong Kong: a cross-sectional study
Source: BMC Public Health. 2020 Apr 21;20:543. doi: 10.1186/s12889-020-08686-6 (PMC7175508; doi:10.1186/s12889-020-08686-6)
Supplement: Supplementary file 1 — Additional file 1. Title of data: Multiple imputation analyses of ever-use of waterpipe. Description of data: Sensitivity analysis of ever-use of waterpipe was conducted through multiple imputation using chained equation to handle missing values. [file 12889_2020_8686_MOESM1_ESM.docx]

**Additional file 1. Multiple imputation analyses of ever-use of waterpipe**

|  | Odd Ratio (OR) (95% CI) | |
| --- | --- | --- |
|  | Crude | Adjusted ^b^ |
| **Gender** |  |  |
| Males | REF | REF |
| Females | 0.96 (0.73-1.26) | 1.38(0.99-2.72) |
| **Age** |  |  |
| 18-19 years | REF | REF |
| 20-21 years | 2.35(1.39-3.96)^**^ | 2.43(1.36-4.32)^**^ |
| 22-23 years | 2.96(1.73-5.04)^***^ | 3.01(1.66-5.45)^***^ |
| 24 year or older | 4.48(2.63-7.64)^***^ | 5.69(2.73-11.85)^***^ |
| **Household income** ^a^ |  |  |
| $19,000 or below | REF | REF |
| $20,000-$49,999 | 1.22(0.89-1.69) | 0.96(0.67-1.38) |
| $50,000 or above | 2.34(1.52-3.62)^***^ | 1.52(0.90-2.55) |
| **Qualification of study** | |  |
| Diploma/Undergraduate | REF | REF |
| Postgraduate | 1.35(0.96-1.91) | 0.56(0.31-1.02) |
| **Alcohol consumption** |  |  |
| Never | REF | REF |
| Once a month or less | 5.47(3.09-9.66)^***^ | 4.67(2.65-8.23)^***^ |
| More than monthly | 30.95(17.26-55.50)^***^ | 22.58(12.41-41.10)^***^ |
| **Sensation seeking behaviour** | |  |
| Low (8-18) | REF | REF |
| Medium (19-29) | 2.79(1.88-4.13)^***^ | 2.04(1.32-3.13)^**^ |
| High (30-40) | 8.18(5.01-13.37)^***^ | 4.80(2.76-8.36)^***^ |
| **Loneliness** |  |  |
| Not lonely | REF | REF |
| Lonely (≥6) | 0.71(0.55-0.92)^*^ | 0.73(0.53-0.98)^*^ |

Key: ^a^ US$ 1=HK$ 7.82; ^b^ Adjusted for all variables listed in the table (i.e., gender, age, household income, qualification of study, alcohol consumption, sensation seeking behaviour and loneliness); ^*^ *p* < 0.05, ^**^*p* < 0.01, ^***^*p* < 0.001.
