# Supplementary material for: Waterpipe smoking among university students in Hong Kong: a cross-sectional study
Source: BMC Public Health. 2020 Apr 21;20:543. doi: 10.1186/s12889-020-08686-6 (PMC7175508; doi:10.1186/s12889-020-08686-6)
Supplement: Supplementary file 2 — Additional file 2. Title of data: Multiple imputation analyses of smoking status. Description of data: Sensitivity analysis of smoking status was conducted through multiple imputation using chained equation to handle missing values. [file 12889_2020_8686_MOESM2_ESM.docx]

**Table S2. Multiple imputation analyses of smoking status**

|  | Waterpipe only | | Cigarette only | | Dual smoking | |
| --- | --- | --- | --- | --- | --- | --- |
|  | Relative risk ratio (RR) (95% CI) | | Relative risk ratio (RR) (95% CI) | | Relative risk ratio (RR) (95% CI) | |
|  | Crude | Adjusted ^b^ | Crude | Adjusted ^b^ | Crude | Adjusted ^b^ |
| **Gender** |  |  |  |  |  |  |
| Males | REF | REF | REF | REF | REF | REF |
| Females | 1.50 (0.99-2.27) | 1.95(1.24-3.05)^**^ | 0.83(0.543-1.26) | 1.06(0.68-1.65) | 0.67(0.47-0.94)^*^ | 1.05(0.69-1.60) |
| **Age** |  |  |  |  |  |  |
| 18-19 years | REF | REF | REF | REF | REF | REF |
| 20-21 years | 2.03(1.05-3.90)^*^ | 2.06(1.03-4.10)^*^ | 1.01(0.45-2.27) | 1.02(0.46-2.26) | 2.90(1.28-6.57)^*^ | 3.12 (1.27-7.70)^*^ |
| 22-23 years | 2.51(1.28-4.90)^**^ | 2.64(1.29-5.41)^**^ | 2.28(1.05-4.93)^*^ | 2.02(0.94 -4.36) | 4.30(1.89-9.79)^**^ | 4.39(1.76-10.97)^**^ |
| 24 year or older | 2.64(1.30-5.36)^**^ | 3.78(1.52-9.39)^**^ | 5.36(2.54-11.35)^***^ | 3.12 (1.23-7.95)^*^ | 10.57(4.71-23.75)^***^ | 12.24(4.26-35.19)^***^ |
| **Household income** ^a^ |  |  |  |  |  |  |
| $19,000 or below | REF | REF | REF | REF | REF | REF |
| $20,000-$49,999 | 1.17(0.74-1.83) | 0.87(0.54-1.40) | 0.60(0.37-0.98)^*^ | 0.62(0.37-1.03) | 1.16(0.76-1.75) | 0.91(0.56-1.47) |
| $50,000 or above | 2.50(1.42-4.41)^**^ | 1.66(0.88-3.15) | 0.66(0.30-1.46) | 0.54(0.24-1.25) | 2.03(1.17-3.51)^*^ | 1.07(0.55-2.07) |
| **Qualification of study** | |  |  |  |  |  |
| Diploma/Undergraduate | REF | REF | REF | REF | REF | REF |
| Postgraduate | 1.01(0.57-1.79) | 0.57(0.24-1.33) | 4.14(2.63-6.51)^***^ | 2.16(1.03-4.51)^*^ | 2.36(1.54-3.59)^***^ | 0.75(0.36-1.58) |
| **Alcohol consumption** |  |  |  |  |  |  |
| Never | REF | REF | REF | REF | REF | REF |
| Once a month or less | 9.59(3.84-23.99)^***^ | 8.13(3.24-20.37)^***^ | 1.69(1.02-2.82)^*^ | 1.71(1.01-2.88)^*^ | 3.58(1.73-7.37)^**^ | 3.21(1.56-6.60)^**^ |
| More than monthly | 36.24(14.14-92.90)^***^ | 27.97(10.68-73.26)^***^ | 4.23(2.39-7.49)^***^ | 3.20(1.62-6.32)^**^ | 30.00(16.33-55.12)^***^ | 26.94(12.90-56.27)^***^ |
| **Sensation seeking behaviour** | |  |  |  |  |  |
| Low (8-18) | REF | REF | REF | REF | REF | REF |
| Medium (19-29) | 2.80(1.64-4.78)^***^ | 2.24(1.27-3.94)^**^ | 1.81(1.05-3.10)^*^ | 1.59(0.91-2.78) | 3.06(1.77-5.29)^***^ | 2.01(1.10-3.66)^*^ |
| High (30-40) | 6.57(3.26-13.22)^***^ | 4.84(2.24-10.44)^***^ | 5.36(2.63-10.90)^***^ | 4.91(2.27-10.63)^***^ | 14.60(7.68-27.74)^***^ | 7.86(3.78-16.36)^***^ |
| **Loneliness** |  |  |  |  |  |  |
| Not lonely | REF | REF | REF | REF | REF | REF |
| Lonely (≥6) | 0.68(0.48-0.98)^*^ | 0.69(0.47-1.02) | 1.43(0.93-2.21) | 1.39(0.88-2.18) | 0.78(0.56-1.10) | 0.84(0.57-1.26) |

Key: ^a^ US$ 1=HK$ 7.82; ^b^ Adjusted for all variables listed in the table (i.e., gender, age, household income, qualification of study, alcohol consumption, sensation seeking behaviour and loneliness); ^*^ *p* < 0.05, ^**^*p* < 0.01, ^***^*p* < 0.001.
